# Oral Anti-Inflammatory and Symbiotic Effects of Fermented Lingonberry Juice—Potential Benefits in IBD

**DOI:** 10.3390/nu16172896

**Published:** 2024-08-29

**Authors:** Pirjo Pärnänen, Ismo T. Räisänen, Timo Sorsa

**Affiliations:** 1 Department of Oral and Maxillofacial Diseases, Head and Neck Center, Faculty of Medicine, University of Helsinki and Helsinki University Hospital, 00290 Helsinki, Finland; 2 Division of Oral Diseases, Department of Dental Medicine, Karolinska Institutet, 171 77 Stockholm, Sweden

**Keywords:** fermented lingonberry juice, oral–gut interactions, inflammatory bowel disease, microbial dysbiosis

## Abstract

Microbial dysbiosis may manifest as inflammation both orally and in the gastrointestinal tract. Altered oral and gut microbiota composition and decreased diversity have been shown in inflammatory bowel disease (IBD) and periodontal disease (PD). Recent studies have verified transmission of oral opportunistic microbes to the gut. Prebiotics, probiotics, or dietary interventions are suggested to alleviate IBD symptoms in addition to medicinal treatment. Lingonberries contain multiple bioactive molecules, phenolics, which have a broad spectrum of effects, including antimicrobial, anti-inflammatory, antioxidant, anti-proteolytic, and anti-cancer properties. An all-natural product, fermented lingonberry juice (FLJ), is discussed as a potential natural anti-inflammatory substance. FLJ has been shown in clinical human trials to promote the growth of oral lactobacilli, and inhibit growth of the opportunistic oral pathogens *Candida*, *Streptococcus mutans*, and periodontopathogens, and decrease inflammation, oral destructive proteolysis (aMMP-8), and dental microbial plaque load. Lactobacilli are probiotic and considered also beneficial for gut health. Considering the positive outcome of these oral studies and the fact that FLJ may be swallowed safely, it might be beneficial also for the gut mucosa by balancing the microbiota and reducing proteolytic inflammation.

## 1. Introduction

We introduce lingonberries as a potential prebiotic and anti-inflammatory substance that might have positive effects also in the gut. We present existing evidence of their in vivo anti-inflammatory, antimicrobial, and anti-proteolytic oral effects. Oral–gut interactions and similarities in microbiome composition and inflammation are considered with IBD as an example of a gut inflammatory condition which causes also oral lesions.

The link between the composition of oral and gut microbiota and gastrointestinal inflammation is an unsettled issue and has been increasingly studied in humans [1,2]. The gut microbiota interact with the host metabolism in multiple ways: tailoring immune responses, and modulation of intestinal mucosa and its permeability. Opportunistic microbes in dysbiotic microbiota may cause degradation of mucosal epithelial extracellular matrix, junctional, and basement membrane proteins, such as collagen, cadherin, occludin, claudin, and laminin. This loss of mucosal barrier integrity together with microbial-triggered host innate immune response may have, when prolonged, even autoimmune or metabolic consequences and predispose to cancer development. The alterations in gut microbiota composition are linked to several diseases, e.g., IBD, asthma, arthritis, obesity, diabetes, and cardiovascular diseases [3,4,5,6,7,8] and even psychiatric disorders [9]. Intestinal microbial dysbiosis has most frequently been determined by the *Firmicutes*/*Bacteroidetes* ratio. Interestingly, the gut microbiota composition as a pathogenic factor in obesity has been proposed. Microbial-targeted therapies—including probiotics, prebiotics, and synbiotics may be beneficial in obesity management [10]. Most studies of the microbiome in IBD have been focused on bacteria, but the role of fungi in microbial dysbiosis has gained interest recently. The increase in *Candida* yeasts in the intestinal microbiota with IBD [11,12] and obesity [5] has been shown.

The objective of this study is to highlight the similarities of microbial dysbiosis and its inflammatory consequences in IBD both orally and in the gut. Microbial dysbiosis and consequent mucosal barrier disintegration via proteolysis and inflammation are proposed as key factors in onset of IBD. As novel targeted IBD medications still wait for results from clinical trials, fermented lingonberry juice is proposed as a safe and clinically studied alternative.

## 2. Lingonberries

Natural substances as health promoters have gained growing interest. They can be a safe alternative as local antimicrobials in the sense that they do not abolish microbiota totally. They aid as prebiotics to guide the microbiota to a healthier composition with a broader spectrum of beneficial microbes. Polyphenols have multiple beneficial effects on cardiometabolic diseases [7]. Lingonberries (*Vaccinium vitis idaea* L.) are wild evergreen shrubs that grow in the northern hemisphere. Multiple fractions isolated from the berries have been studied in vitro and in vivo and proven to have versatile positive effects: antimicrobial, anti-inflammatory, anti-proteolytic (aMMP-8), antioxidant, anticarcinogenic, and cardiovascular effects (Table 1).

Lingonberries contain a unique set of phenolic substances, with antimicrobial, anti-inflammatory, antioxidant, anti-proteolytic, and anti-cancer activities [24]. Lingonberry polyphenols are categorized in the following classes: flavonoids [anthocyanins, flavonols (quercetin), flavanols (catechins), and phenolic polymers (proanthocyanidins)], phenolic acids, lignans, and stilbenes (resveratrol). Anti-inflammatory effects have been found with polyphenol and anthocyanin fractions, proanthocyanidins, kaempferol, and resveratrol; antimicrobial (pathogens) and probiotic growth-enhancing substances from polyphenols (non-fractioned) and anthocyanins.

These bioactive molecules affect the microbiota, gut mucosa, and eventually the inflammatory and metabolic state in a mouse model of obesity [18], low-grade inflammation in high-fat diet mice [16], and glycemic and lipidemic responses in humans [20]. Lingonberry flavonoids decrease the *Firmicute*/*Bacteroidetes* ratio in Apoe^−/−^ mice, which is considered beneficial to prevent overweight [21]. Lingonberries have also been shown to alter the gut microbiota, improve metabolic functions, reduce gut inflammatory properties, and improve brain function in Apoe^−/−^ mice fed a high-fat diet [22]. It should be kept in mind that the extraction methods and composition of the lingonberry fractions affect the results obtained.

**Figure 1:**
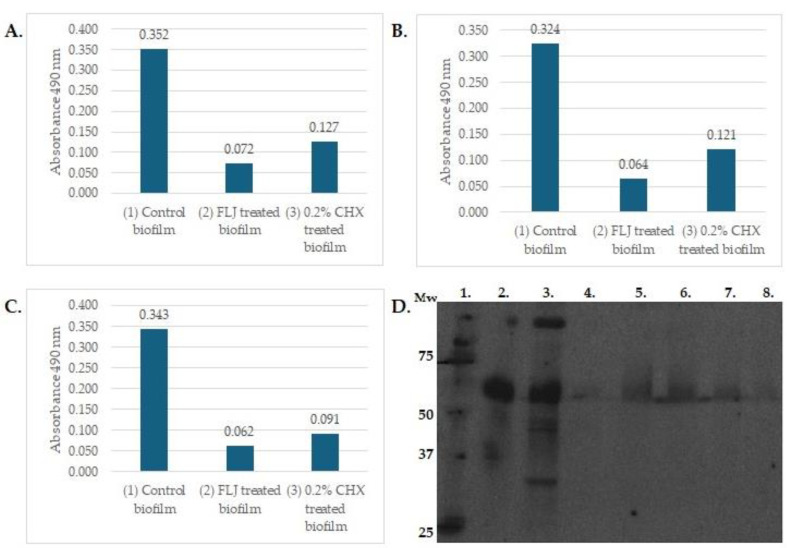
The effects of 24 h incubation with FLJ (0.1 g/mL, 1:1 dilution) on 72 h maturated biofilm formation of *E. faecalis* 29212 (**A**), *E. coli* ATCC 25922 (**B**), and 24 h maturated biofilm of *C. glabrata* CCUG 32725 (**C**) were assessed by XTT assay as previously described [27]. Y-axis: absorbance with 490 nm, X-axis: (1) untreated control biofilm, (2) FLJ-treated biofilm, (3) 0.2% CHX-treated biofilm. High absorbance indicates viable cells in biofilm. The Western blot assay represented effects of FLJ on the activation of proMMP-8 (**D**) and was carried out as previously described [19]. Lanes: 1. Mw standard in kDa, 2. proMMP-8, 3. proMMP-8 activated with >50 kDa *C. glabrata* cell wall protease fraction, lanes 4.–8. proMMP-8+ *C. glabrata* > 50 kDa fraction+ FLJ (lane 4) 40 µL, (lane 5) 5 µL, (lane 6) 10 µL, (lane 7) 20 µL, (lane 8) 40 µ. Lanes 2.–4. incubated 0 h; lanes 5.–8. incubated o/n.

A natural mouthrinse based on fermented lingonberry juice, FLJ, has been developed and targeted for safe oral use by reducing the amount of naturally occurring sugars by fermentation [13]. FLJ has been proven to inhibit growth of typical oral opportunistic bacterial and yeast growth, including *Streptococci*, e.g., *Streptococcus mutans*, *Candida*, and the periodontopathogens *Fusobacterium nucleatus* and *Porphyromonas gingivalis*. Fermentation increases the bioavailability and bioactivity of polyphenolic compounds by enzymatic conversion of large molecules to small ones. This improves absorption in the gut. In clinical human studies, it was found that FLJ has multiple beneficial effects in the oral cavity: it decreases proteolytic inflammation (mouthrinse aMMP-8 levels) and microbial load by inhibiting potential pathogen growth but allows probiotic lactobacilli to survive and increase in numbers [13,14,15]. The total number of participants was 65 in these studies. FLJ (Lingora^®^, Berries United, Finland; 0.1 g/mL, dw/v) was used as an oral rinse 1–2 daily for 30 s for a period of 1–2 weeks [13,14]—6 months [15,23] with 2–4 weeks [13,14]—12 months [15,23] follow-up. Oral indexes and active matrix metalloproteases (aMMP-8) levels were recorded. Additionally, microbial saline rinse samples [14,15] and saliva samples were collected [23]. The clinical outcome in these studies can be seen as improved oral health, reduced microbial opportunistic microbial and proteolytic load, reduced dental plaque and bleeding of the gums, and decreased periodontal inflammation. These effects occurred irrespective of diet or oral homecare habits, contrary to previous results from other studies on diet effects on the microbiota [25]. It also increases salivary flow, which is an important factor as a protective agent for the oral mucosa and dentition [23]. Saliva plays an important role in maintaining oral symbiosis [26], where the host microbiota are kept in healthy balance. Effects of fermented lingonberry juice on inhibition of *E. coli*, *E. faecalis*, and *C. glabrata* biofilm formation, compared to chlorhexidine, and inhibition of matrix metalloprotease-8 activation by *C. glabrata* cell wall proteases may be seen in Figure 1; methods are those described by Pärnänen et al. [19] and Ramage et al. [27].

## 3. Inflammatory Bowel Disease

IBD is a heterogenous group of disorders with excessive inflammation in the intestinal mucosa. The etiology of IBD is unclear and complex, but genetic susceptibility (IBD1, MHC allele HLADRB1*0103, and ABCB1 genes) and some triggering factors, such as infectious agents, diet, drugs, smoking, and altered microbiota, have been suggested [28]. Decreased *Firmicutes*/*Bacteroidetes* ratio has been shown in IBD [12]. The two main classes of IBD are ulcerative colitis (UC) and Crohn’s disease (CD). UC affects mainly the lower intestines; Crohn’s disease causes lesions in the whole gastrointestinal tract. IBD presents also extraintestinal symptoms, such as diarrhea, weight loss, or malnutrition. Prevalence of oral symptoms is 5–50%, which may occur before, during the active phase, or in the remission phase of IBD. Oral manifestations may be the first sign of IBD in 60% of cases. Oral symptoms may be more severe in active IBD, but up to 30% of patients suffer from oral symptoms during the remission phase of IBD. A part of the oral symptoms may be due to intestinal malabsorption [28]. Specific oral manifestations of ulcerative colitis are aphthous stomatitis and pyostomatitis vegetans, and in Crohn’s disease, indurated tag-like lesions, cobblestoning, and mucogingivitis. Non-specific lesions include: atrophic glossitis, burning mouth syndrome, angular cheilitis, dry mouth, taste change, halitosis, periodontitis, recurrent aphthous stomatitis (RAS), persistent submandibular lymphadenopathy, decreased saliva production, halitosis, caries, gingivitis, candidiasis, odynophagia, dysphagia, enlarged intrinsic salivary glands, perioral erythema, recurrent oral abscesses, glossitis, pale mucosa, lichen planus, and metallic dysgeusia. Treatments of these lesions vary from local analgesia and topical agents to systemic steroids and immunosuppressive medications [29], which may cause oral side-effects to be considered. Treatment of CD is more difficult, because the mucosal lesions are deeper than in UC.

Prebiotics, probiotics, or dietary interventions have been proposed for alleviating symptoms in IBD [30]. Probiotics have been proposed to act in several ways to alleviate symptoms of IBD by: (i) reducing oxidative stress which is thought to be one triggering factor in the onset of IBD, (ii) strengthening the intestinal barrier, (iii) increasing diversity and abundance of symbiotic bacteria and decreasing opportunistic pathogen load, (iii) regulating immune response to secrete less proinflammatory and more anti-inflammatory cytokines. Tens of clinical studies have been conducted with bifidobacteria, lactobacilli, *E-coli*, *S. boulardii*, and different combinations on induction and maintenance of UC and CD but only in UC is there strong evidence that *Escherichia coli* Nissle 1917 and *Bifidobacterium* and VSL#3 aid in the remission process [31]. Novel targeted therapies to manage IBD are currently being studied, such as Janus kinase inhibitors, anti-integrins, sphingosine-1-phosphate receptor modulators, anti-interleukin-34-antibodies, and stem cells [32], but also anti-inflammatory natural products have been suggested [33]. Dietary polyphenols are enzymatically transformed into metabolites, underlining the importance of a balanced microbiome which is beneficial for health [34]. Human studies on natural products, e.g., Curcumin, Mastiha, *Boswellia serrata*, and *Artemisia absinthium*, have been limited, sample sizes small, and the phytochemical bioavailability, optimal doses, and safety issues may have been unsolved [35].

## 4. Oral–Gut Interactions

The oral cavity and gut have a bidirectional interaction and the oral cavity may be seen as a window to gut health and disease. It has been shown that there are distinct differences in oral and gut microbiota composition in IBD patients compared to healthy controls [36]. The dysbiotic gut microbiome in IBD has been shown to be colonized by opportunistic microbes from the oral cavity, and the oral microbiota in IBD patients suffering from periodontitis have lower alpha-diversity [37]. Oral inflammatory diseases, such as gingivitis, periodontitis, and stomatitis, have been linked to gastrointestinal diseases, such as IBD [38]. Treatment of periodontal disease has a positive effect on course of disease in IBD. Certain oral periodontal dysbiotic bacteria (e.g., *Porhyromonas gingivalis*) may be swallowed and have been found to cause gut microbiota dysbiosis found in liver cirrhosis, rheumatoid arthritis, or gastrointestinal cancers. Proper oral homecare, periodontal treatment, and pro- or prebiotic supplements are suggested for reducing this inflammatory load [39]. Approximately 60% of oral bacteria are frequently transmitted to feces by shotgun metagenomic analyses, and in certain diseases, such as colorectal cancer and rheumatoid arthritis, the transmission is more frequent than in healthy counterparts [40]. Since over 1000 mL of saliva is produced per day and most of it is swallowed and passed to the intestinal tract, oral dysbiotic microbiota have an opportunity to colonize the intestines to some degree. Lower lysozyme and elevated IL-1β, IL-8, IgA are strongly correlated to salivary microbiota composition; in UC and in CD, increased cytokine and IgA salivary levels have been detected [41]. Dysbiotic periodontal bacteria may manipulate host immune responses resulting in systemic inflammation in susceptible individuals [42]. Neutrophils play a central role in innate immunity against infection and take part in tissue repair. They also secrete metalloproteases (MMPs), myeloperoxidase (MPO), and neutrophile elastase (NE), which may cause tissue damage in prolonged inflammation. Neutrophil extracellular traps (NETs) are an integral defense mechanism against high loads of bacteria viruses and fungi. Increased amounts of NET formation have been recorded in IBD [43]. Elevated levels of neutrophil-derived MMPs, MPO, and NE are also found from oral inflammatory sites. If the inflammation progresses, macrophages are the next regulators. If the inflammation is not resolved, it may become a chronic inflammation with systemic effects [44]. The known translocation of oral bacteria into the gut and the similarity of comparison results at the genus level of oral and gut microbiota suggest that the oral microbiome may be a novel interventional target for manipulating the gut microbiome [45]. Active matrix metalloprotease-8 (aMMP-8) is an inflammatory marker of low-grade inflammation in the oral cavity measured from mouthrinse, saliva, gingival crevicular fluid, or peri-implant sulcular fluid. aMMP-8 is a key biomarker as the driving underlying pathological mechanism in periodontitis and systemic diseases and conditions, such as prediabetes, colorectal cancer, and obesity [46], and maybe in the future useful in diseases affecting the cardiovascular system, cancers, bacteremia, sepsis, diabetes, obesity, meningitis, as well as pancreatitis. Elevated aMMP-8 levels have also been recorded from IBD patients [37,47,48]. Activation of matrix metalloprotease-8 (MMP-8) by opportunistic *Candida glabrata* yeast cell wall proteases to its active form (aMMP-8) has been shown to be inhibited by FLJ [19]. The anti-inflammatory effects and reduced microbial load by FLJ have been clinically verified by aMMP-8 chairside tests in clinical human oral studies [14,15,17].

## 5. Discussion

The oral cavity could be seen as a gateway for pathogens to spread and pose a risk for general health as acute or chronic inflammatory processes. This kind of low-grade inflammation is a stress to the body and immune system. It may manifest as autoimmune diseases, metabolic syndrome, cardiovascular diseases, inflammatory bowel disease, or obesity. Proper microbiota composition is crucial for the gut mucosa to remain intact and act as a protective barrier. Harmful microbes are capable of degrading host mucosal proteins, which might lead to inflammation of the gut or infection/invasion of pathogens. Microbes in the gut are constantly co-playing or battling for nutrients and interact with each other by secreting substances harmful for their rivals; indeed, lactobacilli are known to secrete antifungal peptides [49]. Lactobacilli are considered probiotic, and increased amounts could occupy the gut mucosa and cut off living space and nutrients from potential pathogens thus limiting their overgrowth. Although proven to be beneficial, commercially produced probiotics have a problem: they may not maintain their viability when reaching the gut. Additionally, it is a challenge to choose which probiotic strain would have the most beneficial properties. Many of the oral inflammatory symptoms found in IBD could be relieved by FLJ as proven in clinical oral human studies [14,15,17]. FLJ may be swallowed safely, the sugar content has been decreased, and it has no observed side-effects or interactions with medications [14,15,17]. In addition, the lactobacilli numbers remained elevated (measured after ½ year washout period [15]. It might be interesting to study if this kind of prebiotic approach could be effective in modifying the gut microbiota to a more symbiotic, health-promoting composition and indirectly decrease low-grade inflammation and risk for gut or overweight problems. FLJ could be used as an aid to alleviate IBD symptoms by balancing the microbiota and reducing inflammation both orally and intestinally. There is growing evidence that dietary polyphenols may alleviate inflammation in IBD [50]. The role of yeasts in the pathomechanism of inflammatory diseases has been a less noticed topic and more detailed studies of bacteria, yeasts, and viruses at strain level are warranted. There are only a few human clinical studies of lingonberry, but now would be the time for personalized dietary interventions and to study the effect of fermented lingonberry juice bioactive polyphenol compounds in aiding the indigenous lactobacilli of the host to thrive better. This may support health in decreasing low-grade inflammation also in the gastrointestinal tract, and maybe prevent or alleviate IBD secondary negative metabolic consequences in the host.

## Figures and Tables

**Table 1:** Effects of lingonberries.

Examples of Bioactivities of Lingonberries		
Antimicrobial	In vivo human studies	[13,14,15]
Anti-inflammatory and antioxidant	In vivo human studies In vitro mouse study	[13,14,15,16,17] [18]
Anti-proteolytic (aMMP-8 ↓)	In vitro and in vivo human study	[14,19]
Glucose, insulin, free fatty acids	In vivo human study In vivo mouse study	[20] [21]
Gut microbiota	In vitro mouse study	[22]
Saliva	In vivo human study	[23]

↓ Decreased aMMP-8 enzyme levels.

## Data Availability

The raw data supporting the conclusions of this article will be made available by the authors on request.
